# P-476. Risk Factors for Mortality in Children and Young Adults with Carbapenem-Resistant Gram-Negative Infections

**DOI:** 10.1093/ofid/ofaf695.691

**Published:** 2026-01-11

**Authors:** Angelique E Boutzoukas, Jiahe Tian, Lauren Komarow, Lizhao Ge, Minggui Wang, Cesar A Arias, Thamer Alenazi, Zhengyin Liu, David L Paterson, Michael J Satlin, Yohei Doi, Vance G Fowler, David van Duin

**Affiliations:** Duke University/Duke Clinical Research Institute, Raleigh, NC; The Biostatistics Center, George Washington University, Rockville, Maryland; George Washington University, Rockville, Maryland; George Washington University, Rockville, Maryland; Institute of Antibiotics, Huashan Hospital, Fudan University, Shanghai, Shanghai, China (People's Republic); Houston Methodist and Weill Cornell Medical College, Houston, TX; King Saud bin Abdulaziz University for Health Sciences, Riyadh, Ar Riyad, Saudi Arabia; Infectious Disease Section, Department of Internal Medicine, Peking Union Medical College Hospital, Beijing, Beijing, China; The University of Queensland, Queensland, Queensland, Australia; Weill Cornell Medicine, New York, NY; University of Pittsburgh, Toyoake, Aichi, Japan; Duke University Medical Center, Durham, NC; University of North Carolina at Chapel Hill, Chapel Hill, NC

## Abstract

**Background:**

Risk factors for mortality among patients with carbapenem-resistant (CR) Gram-negative infections have been extensively studied, however children and younger adults with CR Gram-negative infections are underrepresented in these studies.

**Table 1.**
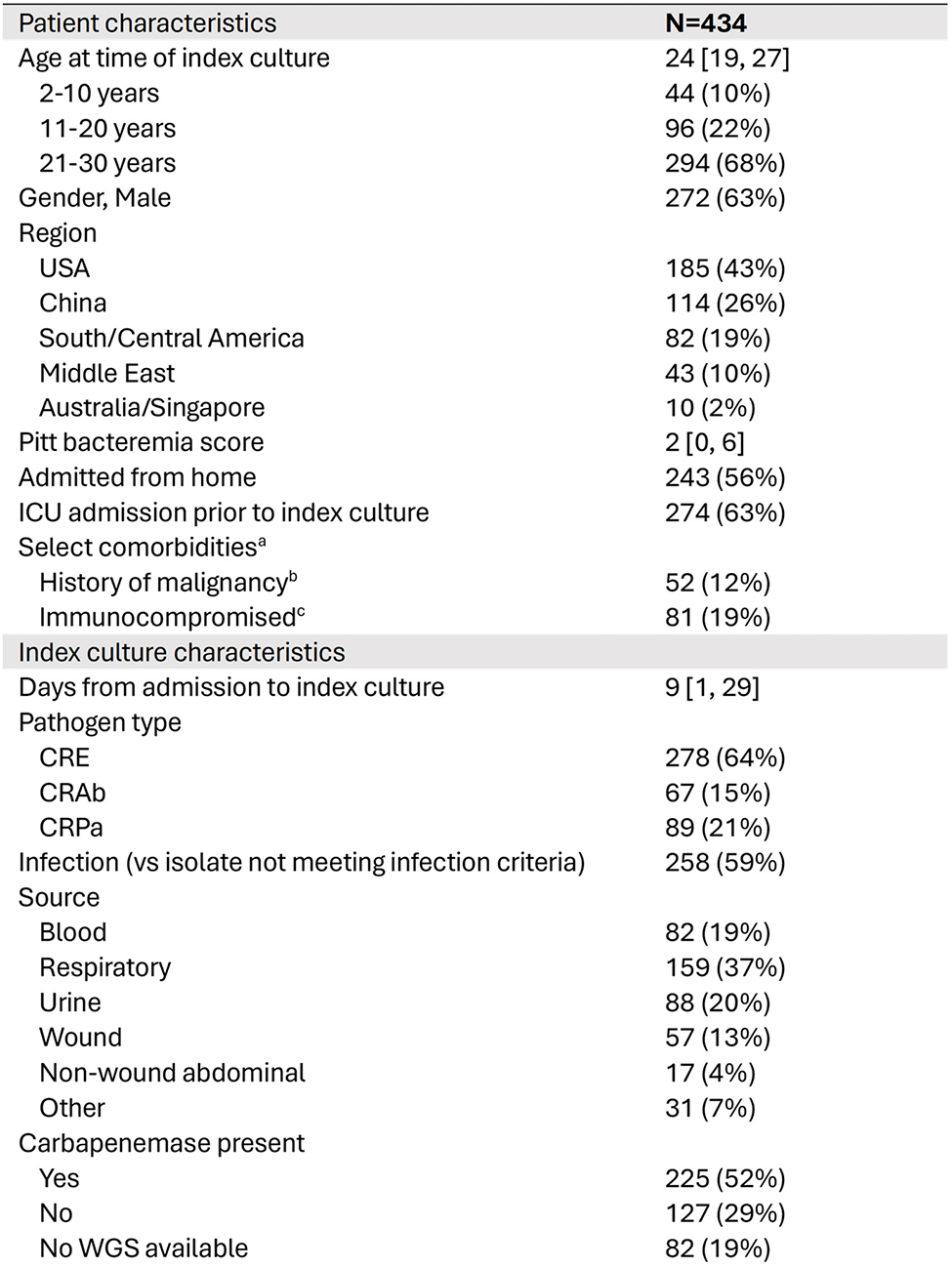
Demographics for included patients aged 2-30 years with carbapenem-resistant isolates (N=434)

**Figure 1.**
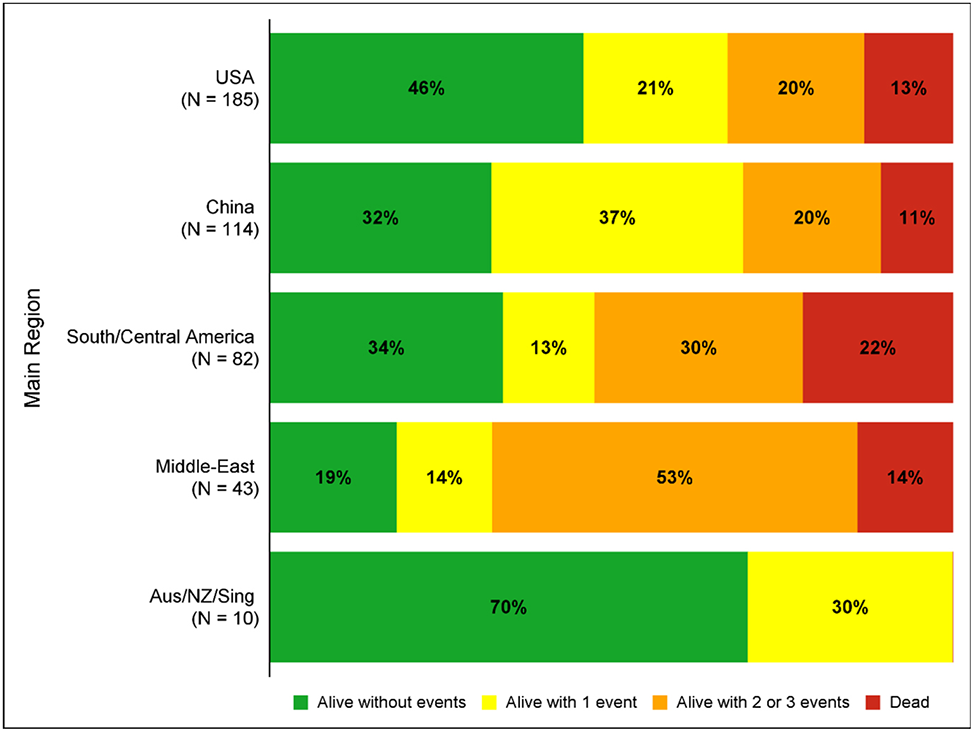
4-category desirability of outcome ranking (DOOR) outcomes in patients aged 2-30 years with carbapenem resistant isolates at 30-days from index culture, by region

The figure shows the distribution of 4-category desirability of outcome ranking at 30 days from the index culture date, by region. The most desirable outcome is to be alive without any events. The least desirable outcome is to be dead at 30 days. The following events were assessed: 1) Lack of clinical response at 30 days, where clinical response is defined as a symptomatic response, no anti-CRE antibiotic, and no relapse within 30 days; 2) not discharged within 30 days or readmitted within 30 days; and 3) renal failure post-culture or C. difficile infection.

**Methods:**

Here, children and young adults 2-30 years old enrolled in one of three MDRO Network studies with a qualifying culture positive for a CR organism (Enterobacterales - CRACKLE/CRACKLE-2; *Acinetobacter baumannii –* SNAP; or *Pseudomonas aeruginosa* – POP) were included. We assessed the relationship between patient characteristics and 30-day mortality (or discharge to hospice) using univariate and multivariable logistic regression models in the full cohort and in the subgroup of patients with isolates that met criteria for infection.

**Table 2.**
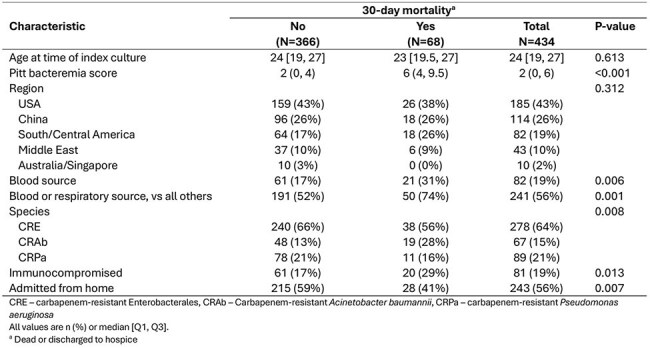
Patient and isolate characteristics by 30-day mortality outcomes

**Table 3.**
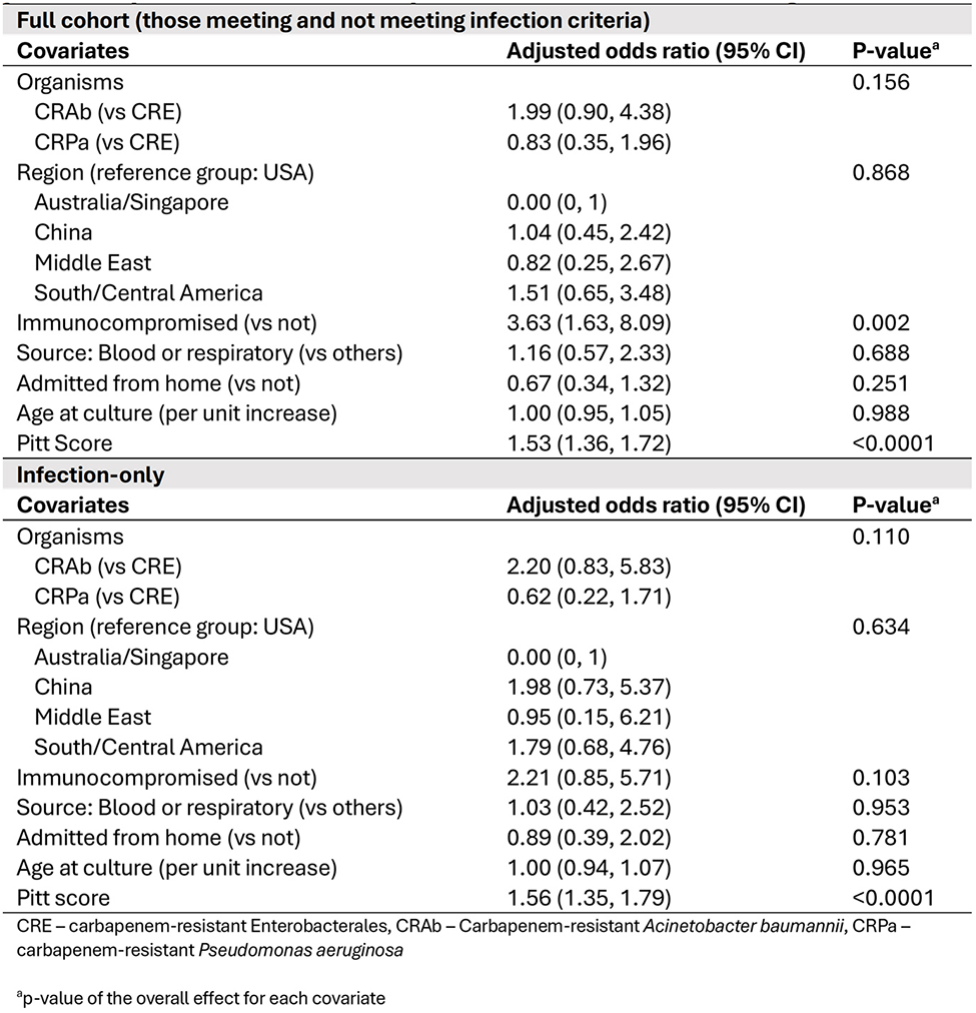
Logistic regression models evaluating predictors of 30-day mortality in 2-30 year old patients with carbapenem-resistant Gram-negative isolates

**Results:**

Across the MDRO Network, 434 eligible patients from 52 sites and 10 countries were included, with a median age of 24 years (Q1 19, Q3 27, Table 1). Of those, 258 (59%) met infection criteria. 30-day outcomes varied by region (p< 0.001, Figure 1). The mortality rate at 30 days was 16% (68/434, including discharge to hospice). On univariate analysis, higher Pitt score, immunocompromised status, admission from a non-home location, pathogen (*A. baumannii* versus others), and source (blood and/or respiratory source versus others) were associated with increased 30-day mortality (Table 2). In multivariable analysis, Pitt score and immunocompromised status were predictors of 30-day mortality (Table 3). For the infection-only subgroup, Pitt score was the sole identified predictor of mortality (Table 3); each one-point increase increased the odds of 30-day mortality by 56% (adjusted odds ratio [aOR] 1.56 [95% CI 1.35, 1.79]).

**Conclusion:**

Acuity of illness, as measured by the Pitt bacteremia score, was identified as a risk factor for mortality in 2–30-year-old patients with CR Gram-negative infections; notably, age was not a predictor of mortality. This contrasts with known predictors of mortality in older adults, including age, organism, region, immunocompromise, and source of infection. Pitt scores may be useful for prognostication of mortality risk in young patients with highly resistant infections.

**Disclosures:**

Angelique E. Boutzoukas, MD, MPH, Elion Therapeutics: Advisor/Consultant|Innoviva Speciality Therapeutics: DSMB Participant Michael J. Satlin, MD, MS, AbbVie: DSMB participant|bioMerieux: Grant/Research Support|Merck: Grant/Research Support|SNIPRBiome: Grant/Research Support Yohei Doi, MD, PHD, GSK: Advisor/Consultant|Meiji Seika Pharma: Advisor/Consultant|Shionogi: Advisor/Consultant|Shionogi: Honoraria Vance G. Fowler, MD, MHS, Affinergy, Janssen, Contrafect: Advisor/Consultant|AstraZeneca; EDE; Basilea: Grant/Research Support|Debiopharm, GSK; Affinium, Basilea,: Advisor/Consultant|Destiny, Amphliphi, Armata, Akagera: Advisor/Consultant|Merck; Contrafect; Karius; Janssen: Grant/Research Support|UpToDate: Royalties|Valanbio: Stock options David van Duin, MD, PhD, British Society for Antimicrobial Chemotherapy: Editor stipend|Merck: Advisor/Consultant|Merck: Grant/Research Support|Pfizer: Advisor/Consultant|Roche: Advisor/Consultant|Shionogi: Advisor/Consultant

